# Mapping Research Trends in AI-Based Tourism and Hospitality Marketing: A Bibliometric and Thematic Review

**DOI:** 10.12688/f1000research.177254.1

**Published:** 2026-02-05

**Authors:** Pankaj Kumar Tyagi, Priyanka Aggarwal, Priyanka Tyagi, Asokan Vasudevan, Premendra Kumar Singh

**Affiliations:** 1 University Institute of Tourism and Hospitality Management, Chandigarh University, Mohali, Punjab, India; 2 Department of Law, Maharaja Agrasen Institute of Management Studies, Delhi, Delhi, India; 3 Faculty of Business and Communications, INTI International University, Negeri Sembilan, Malaysia; 4 Research Fellow, Wekerle Business School, Budapest, Hungary; 5 Centre for Distance & Online Education, Sharda University, Greater Noida, Uttar Pradesh, India

**Keywords:** Tourism, Hospitality, Marketing, Artificial Intelligence, Systematic literature review, Bibliometric Analysis, Thematic Analysis, Process Innovation, SPAR-4-SLR

## Abstract

**Background:**

Artificial intelligence (AI) has fundamentally transformed tourism and hospitality marketing through enhanced data-driven decision-making, personalized customer experiences, and intelligent destination management. However, the field lacks a comprehensive synthesis of its intellectual landscape and thematic evolution, limiting understanding of research trajectories and emerging directions.

**Methods:**

A systematic literature review following the SPAR-4-SLR procedure was conducted on 320 peer-reviewed papers published between 2003 and 2025, sourced from the Scopus database. Publication trends, leading journals, prolific authors, trending areas, and bibliographic coupling of documents and countries were visualized using bibliometric analysis tools (VOSviewer and Biblioshiny). Thematic analysis employed keyword co-occurrence networks to identify emerging research themes.

**Results:**

Academic publications on AI in tourism and hospitality demonstrated a significant surge during 2017–2020, reflecting the industry’s growing emphasis on smart marketing applications. Thematic analysis identified four major research clusters: (i) Digital Influence and Tourist Behaviour Analytics; (ii) AI-Enabled Smart Tourism and Commerce Ecosystems; (iii) Technology-Driven Hospitality and Experience Innovation; and (iv) Data-Driven Decision Making in Predictive Tourism Modelling.

**Conclusions:**

This bibliometric and thematic assessment reveals the evolving intellectual landscape of AI applications in tourism and hospitality marketing, documenting substantive research growth and the emergence of distinct thematic clusters that shape current and future research agendas in this dynamic field.

## 1. Introduction

The tourism and hospitality sector faces a profound digital transformation, with Artificial Intelligence (AI) leading the effort to generate competitive edges in fields such as marketing or customer relationship management. With the advent of machine learning, big data analytics, natural language processing, and algorithmic decision-making technology, the way in which technology is employed for customer acquisition, conversion and retention for tourism and hospitality firms online has been transformed. AI-powered technologies are also embedded in marketing activities such as customer scoring, dynamic pricing, recommendation engines, demand planner forecast, sentiment analysis, and personalized campaign instead of typical strategic and tactical marketing. Prior research has demonstrated that AI may assist firms to enhance marketing effectiveness by handling and analyzing copious amounts of structured and unstructured data (e.g., past purchases, browsing behaviors, social media communications, web reviews, or real-time behavioral cues). Such insights derived from data can help companies in developing more personalized products and personalized promotional messages and also rationalising their pricing strategies for context-based user experiences, leading to an increase in conversion rate, brand loyalty and CLV ( Samala et al., 2022). Negligible AI-based marketing tools in tourism and hospitality sectors where the level of service differentiation is high, are yet even more potent as the characteristics of intangibility, perishability, and information asymmetry is existential through the destination image formation (as a precursor to pleasure), travel intention or motivation (driver towards psychological commitment), as well as perceptions toward services provided by the industry. Despite these opportunities, the growing reliance on AI in tourism and hospitality marketing activities has raised significant questions about ethics, data privacy, transparency of use of algorithms, trust from consumers as well as levels of “human touch” in service interactions ( Kandampully et al., 2023). However, despite the enabling aspects of automation and AI-driven systems in making things more efficient and scalable, an over-dependence on AI might lead to depersonalisation, biased decisions and reduced emotional engagement (critical for providing memorable tourist experiences). Consequently, current literature tries to overcome this kind of trade-off by emphasizing responsible, explainable and trust-based AI use as opposed to technology intelligence versus human-centric marketing. Although there is an abundance of research on AI, tourism and marketing, this literature is fragmented across domains, methodologies and application fields. The retrieved work covers a variety of theoretical underpinnings, methodological approaches and context of studies including social media analytics and sentiment analysis, smart tourism ecosystems, immersive technologies to predictive modeling. While narrative reviews and conceptual papers exist in the literature, a comprehensive and systematically built synthesis on the intellectual roots, publication patterns and emerging thematic clusters of AI tourism and hospitality marketing research is lacking. To fill this gap, the current article systematically investigates AI-based tourism and hospitality marketing research via a bibliometric and thematic review approach within the SPAR-4-SLR protocol framework with advanced bibliometric operations as well as keyword co-occurrence analysis. Through the lens of 320 peer-reviewed articles published from 2003 to 2025, this review paper attempts to offer a comprehensive picture on field development in terms of dominating and emerging research themes and suggestions for future work. Based on these theoretical and empirical gaps, the aims of this article are to answer three research questions (RQs) by reviewing existing literature:

RQ1:
*What is the current state of research in the field of AI-Based Tourism and Hospitality Marketing?*

RQ2:
*What are the emerging themes associated with AI-Based Tourism and Hospitality Marketing?*

## 2. Methodology

The study employed a three-step methodology consisting of the SPAR-4-SLR protocol, bibliometric analysis, and thematic analysis to systematically review research publications. Systematic literature reviews (SLRs) are designed to minimize bias and maximize transparency and reproducibility ( Brignardello-Petersen et al., 2025; Shehata & D’Souza, 2025). SLRs start from an explicit question, eligibility criteria, and a registered or predefined protocol, reducing selective decision-making and reporting bias ( Boell & Cecez-Kecmanovic, 2015; Tranfield et al., 2003). A systematic literature review provides a comprehensive overview of the existing research, addresses gaps, and presents avenues for future studies ( Paul & Criado, 2020; Tranfield et al., 2003). In the first phase, the SPAR-4-SLR protocol is utilised to systematically gather the research papers from the Scopus database. The second step consisted of bibliometric analysis using VOSviewer (version 1.6.8) and Biblioshiny software. The bibliometric review was particularly fit for purpose in this study because it focuses on statistics and trends of a review domain ( Castillo-Vergara et al., 2018). Last, a thematic analysis was conducted through keyword co-occurrence by VOSviewer.

### 2.1 SPAR-4-SLR protocol

The SPAR-4-SLR protocol, proposed by Paul et al., (2021), delineates the methodology’s framework, comprising three stages and six substages:

**2.1.1 Assembling**

***2.1.1.1 Identification***

This study delineates AI-based tourism and hospitality marketing as the focal domain of the review. The research is guided by Research Questions 1 and 2 (presented at the end of the Introduction), which direct the examination of bibliometric patterns, research characteristics, intellectual relationships, thematic structures, and future research agendas within the domain. To achieve these objectives, scholarly publications related to AI-based tourism and hospitality marketing were systematically retrieved from the Scopus database.

***2.1.1.2 Acquisition***

Scopus was selected as the primary database for literature retrieval due to its extensive coverage and reliability, despite the possibility of minor publication time lags ( Paul et al., 2021). Relevant publications within the management and allied disciplines were collected in December 2025, covering the period from 2003 to 2025. A comprehensive search strategy employing Boolean operators was adopted. Consistent with prior review methodologies, the operator “OR” was predominantly used to avoid excessive restriction in retrieval Paul et al., (2021). The search string included the following keywords: “artificial intelligence” OR “AI” OR “machine learning” OR “deep learning” OR “big data” OR “chatbot*” OR “algorithm*” AND “tourism” OR “travel” OR “hospitality” OR “hotel*” OR “destination*” OR “tourism industry” AND “marketing” OR “digital marketing” OR “destination marketing” OR “branding” OR “promotion” OR “advertising” OR “customer engagement”. No temporal restrictions were imposed at the initial stage, resulting in a dataset spanning twenty-three years (2003–2025). The search was conducted across titles, abstracts, and keywords to ensure alignment with the predefined search terms, yielding an initial pool of 2,228 documents ( Paul et al., 2021).

**2.1.2 Arranging**

***2.1.2.1 Organization***

Following the approach recommended by Paul et al., (2021), a structured codebook was developed to systematically record and classify the retrieved studies. The coded variables included citation details, references, journal title, document type, year of publication, citation count, author keywords, and country of origin.

***2.1.2.2 Purification***

To enhance the relevance and quality of the dataset, rigorous inclusion and exclusion criteria were applied. A manual screening process confirmed the absence of duplicate records. The inclusion criteria encompassed: (i) document types such as journal articles, conference papers, reviews, and editorials; (ii) subject areas including Business, Management and Accounting, Social Sciences, Environmental Science, Economics, Econometrics and Finance; and (iii) publications written in English. Documents unrelated to AI-based tourism and hospitality marketing were excluded through a detailed assessment of titles, keywords, abstracts, and, where necessary, introductions. This purification process resulted in the exclusion of 1,908 records. Consequently, a final sample of 320 relevant documents was retained for in-depth analysis, as summarized in Table 1.

**Table 1.** Purification process.

Search criteria	Entry	Results
Search Field	Article title, Abstract, Keywords	
Articles identified through Keywords and Boolean term searches (Scopus Database)	“artificial intelligence” OR “AI” OR “machine learning” OR “deep learning” OR “big data” OR “chatbot*” OR “algorithm*” AND “tourism” OR “travel” OR “hospitality” OR “hotel*” OR “destination*” OR “tourism industry” AND “marketing” OR “digital marketing” OR “destination marketing” OR “branding” OR “promotion” OR “advertising” OR “customer engagement”	2,228
Filter 1	Document type: Articles, Conference Paper, Review, Editorial	1,741
Filter 2	Scopus Categories: Business, Management, and Accounting, Social Sciences, Environmental Science, Economics, Econometrics and Finance	910
Filter 3	Language: English	867
Filter 4	Documents not pertinent to AI and Tourism (irrelevant papers) – evaluated based on the title, keywords, abstract, and, when applicable, the introduction	547
Full-text Articles included in qualitative analysis	Articles were selected after reading the complete research papers	320

Source: Author’s own.

**2.1.3 Assessing**

***2.1.3.1 Evaluation***

In line with the framework proposed by Paul et al., (2021), the analysis of the selected studies was primarily conducted using bibliometric techniques. To comprehensively map the intellectual structure and thematic evolution of the literature, multiple analytical methods were employed, including bibliographic coupling, cluster analysis, and keyword co-occurrence analysis. In addition to these techniques, sentiment analysis was performed using R software to capture the prevailing and emerging sentiments within the AI-based tourism and hospitality marketing literature and to infer potential future research directions.

***2.1.3.2 Reporting***

The findings of the systematic literature review are presented through a combination of narrative discussion and visual representations. These outputs emphasize the classification, structure, and thematic organization of the existing body of knowledge. The study concludes with a critical discussion of its limitations, thereby providing transparency and context for the interpretation of the results.

### 2.2 Bibliometric and thematic analysis

This study adopts a bibliometric and thematic approach to systematically review the literature on AI-based tourism and hospitality marketing, utilizing the VOSviewer and Biblioshiny software packages. Bibliometric analysis has indeed become highly prominent and methodologically sophisticated in business and management, largely because of powerful tools and broader access to Scopus and Web of Science ( Caputo & Kargina, 2022; Donthu et al., 2021). In the present study, VOSviewer and Biblioshiny were employed to generate comprehensive descriptive and relational insights, including publication trends, three-field plots, identification of influential publications, country-level and document-level bibliographic coupling, keyword co-occurrence networks, thematic evolution, trend analysis, and WordCloud visualizations. Together, these analyses provide a structured and multidimensional understanding of the intellectual foundations, thematic clusters, and emerging trajectories in AI-based tourism and hospitality marketing research.

## 3. Findings

### 3.1 Annual scientific production

**Figure 1.**
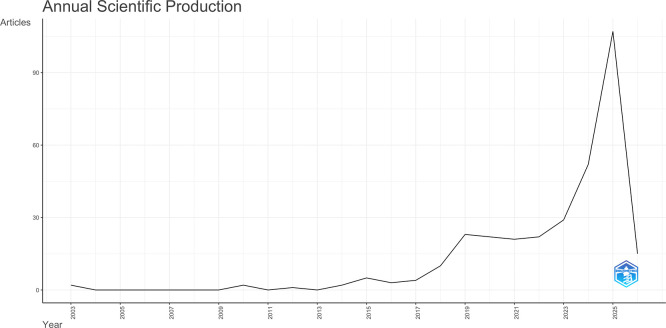
Annual scientific production (Source: Biblioshiny).

The search of the Scopus database identified 320 publications concerning AI-driven tourism and hospitality marketing, with the earliest publication dating back to 2003. Based on the analysis of the publication structures, it is evident that the volume of literature has significantly increased over the past four years ( Figure 1). The expanding corpus of literature establishing the legitimacy of AI-driven tourism and hospitality marketing as a scholarly discipline.

### 3.2 Three-fields plot

**Figure 2.**
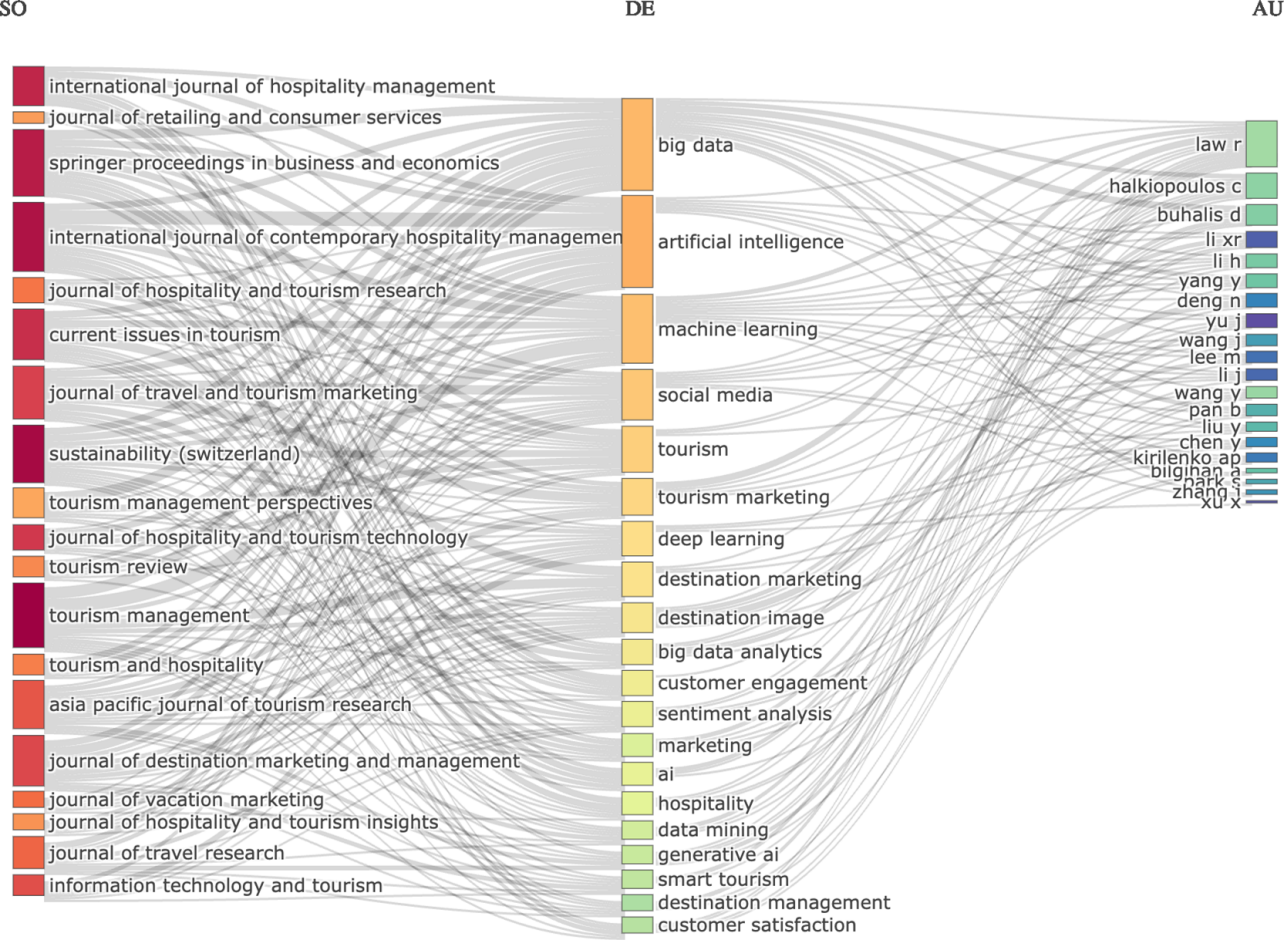
Three-fields plot (Sources, keywords and authors) (Source: Biblioshiny).

The three field diagram has shown the conceptual framework of research themes on AI, tourism and marketing with links among sources (journal names), descriptors (author keywords), and authors ( Figure 2). This chart is based on the most popular Sankey-diagrams. The three components are visualized with the grey links representing their connection, from the keywords to countries and journal titles. Sizes of the boxes reflect the frequencies ( Riehmann et al., 2005). As a result, the size of every rectangle in every list shows the number of documents to that element. The reference dimension reveals that the International Journal of Hospitality Management, International Journal of Contemporary Hospitality Management, Tourism Management, Tourism Tourism Management Perspectives, Journal of Travel & Tourism Marketing and Information Technology & Tourism are identified as key publications outlets by showing that hospitality- tourism marketing and technologyfocused research provides the foundation to this social universe. From the descriptor field, it can be seen that technology-oriented topics such as artificial intelligence (AI), big data, machine learning, deep learning and generative AI are dominating the research scene and marketing-based ideas such as tourism marketing, destination marketing, destination image communication with customers and customer satisfaction complete this picture. It also shows that for the tourism industry, social media sentiment analysis and big data analytics should be increasingly focused on the move towards more personal marketing strategies. In conclusion, the solid nodes linking sources to keywords reveal a fast growing interdisciplinary field of research to reshape marketing strategy, destination management and customer experience in tourism and hospitality industry by AI-driven technologies.

### 3.3 Most influential publications

**Figure 3.**
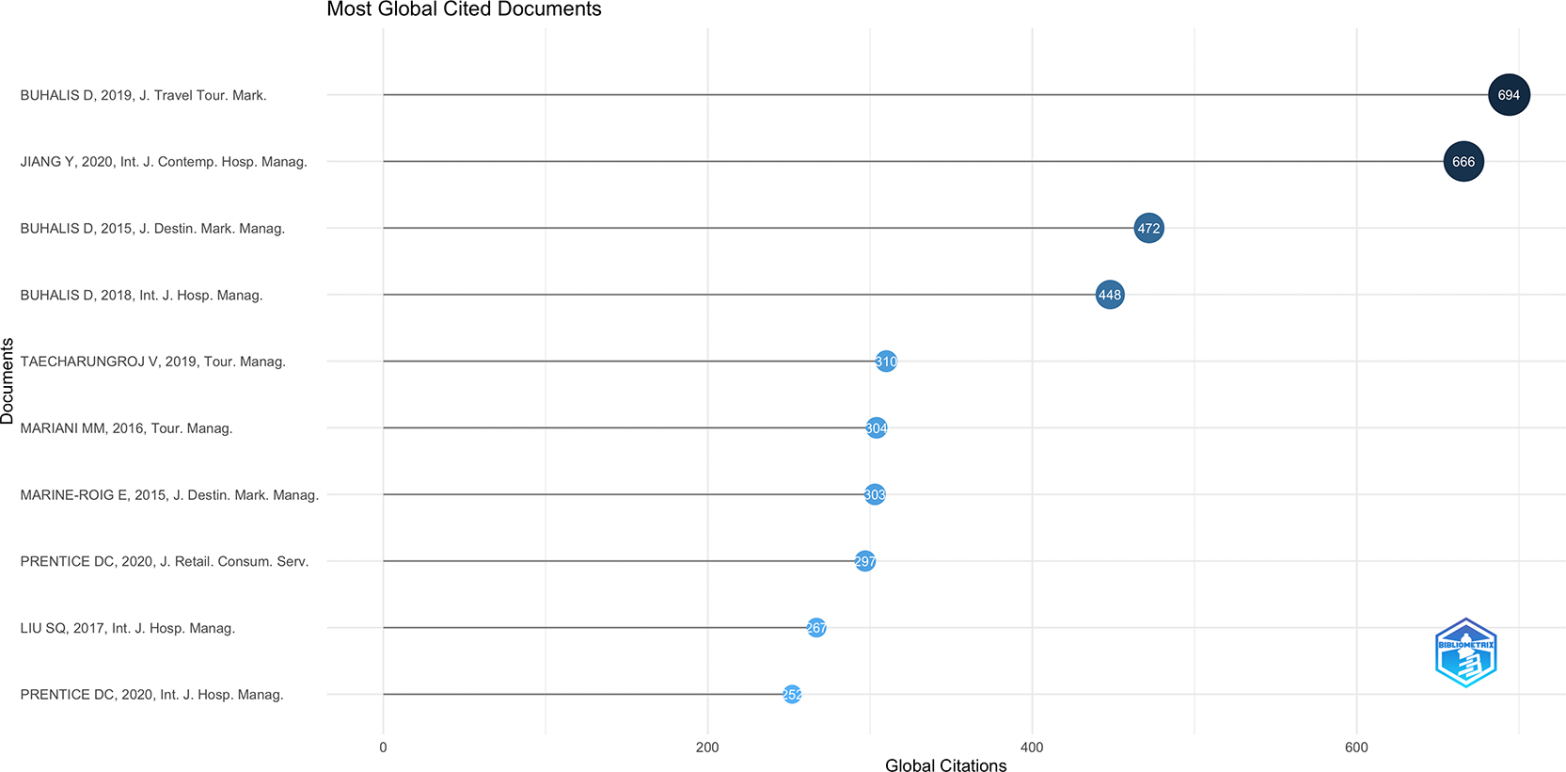
Most influential publications (Source: Biblioshiny).

Figure 3 reveals top 25 influential articles according to the number of global citations. Global citation count is an index tracking the overall number of citations from databases, including the whole of Scopus ( Garfield, 2003). The analysis also identifies emerging leading in works to be located by Buhalis, as the articles of which he was author/co-author dominated highly positioned ranks, demonstrating his trailblazing role in shaping technology and AI-based tourism academic research. The most influential study is particularly Buhalis’s ( Buhalis et al., 2019) paper from the Journal of Travel & Tourism Marketing—in terms of global citations—followed closely by Jiang & Wen, (2020) in the International Journal of Contemporary Hospitality Management.

### 3.4 Word cloud

**Figure 4.**
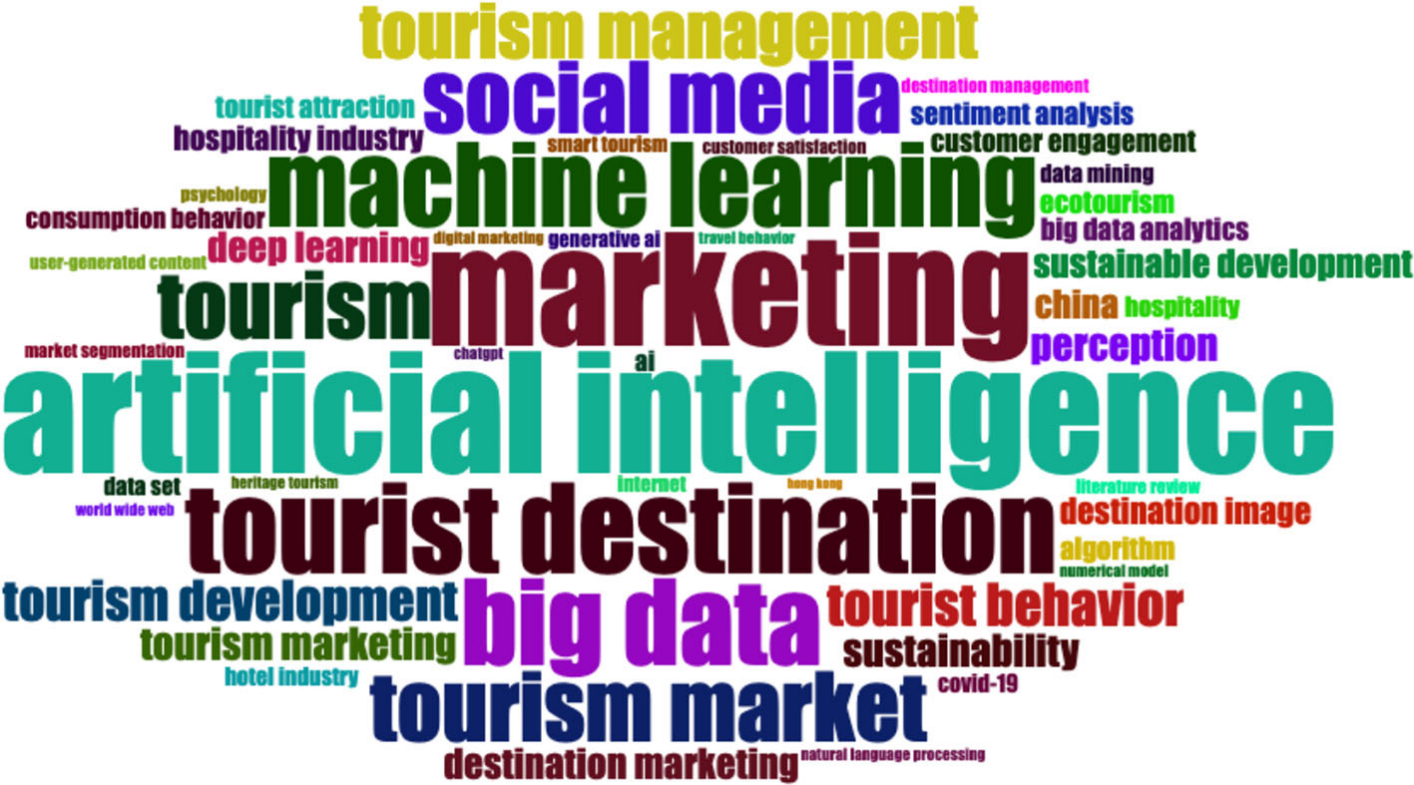
Word cloud (Source: Biblioshiny).

The word cloud indicates the central academic research topics on artificial intelligence for tourism marketing—artificial intelligence, marketing, machine learning, big data and tourist destination ( Figure 4). The abundant use of social media and the tourism management perspective emphasized the significance of digital ecosystems and data-driven action in tourist domains. Instead, terms such as “tourist behaviour,” “destination image” or “customer engagement” show a clear concern about understanding and enhancing the experience of consumers. Overall, the visualization suggests a growing interest in the use of AI-enhanced and analytics-enabled marketing strategies to support destination competitive advantage development and tourism market performance.

### 3.5 Trend topics

**Figure 5.**
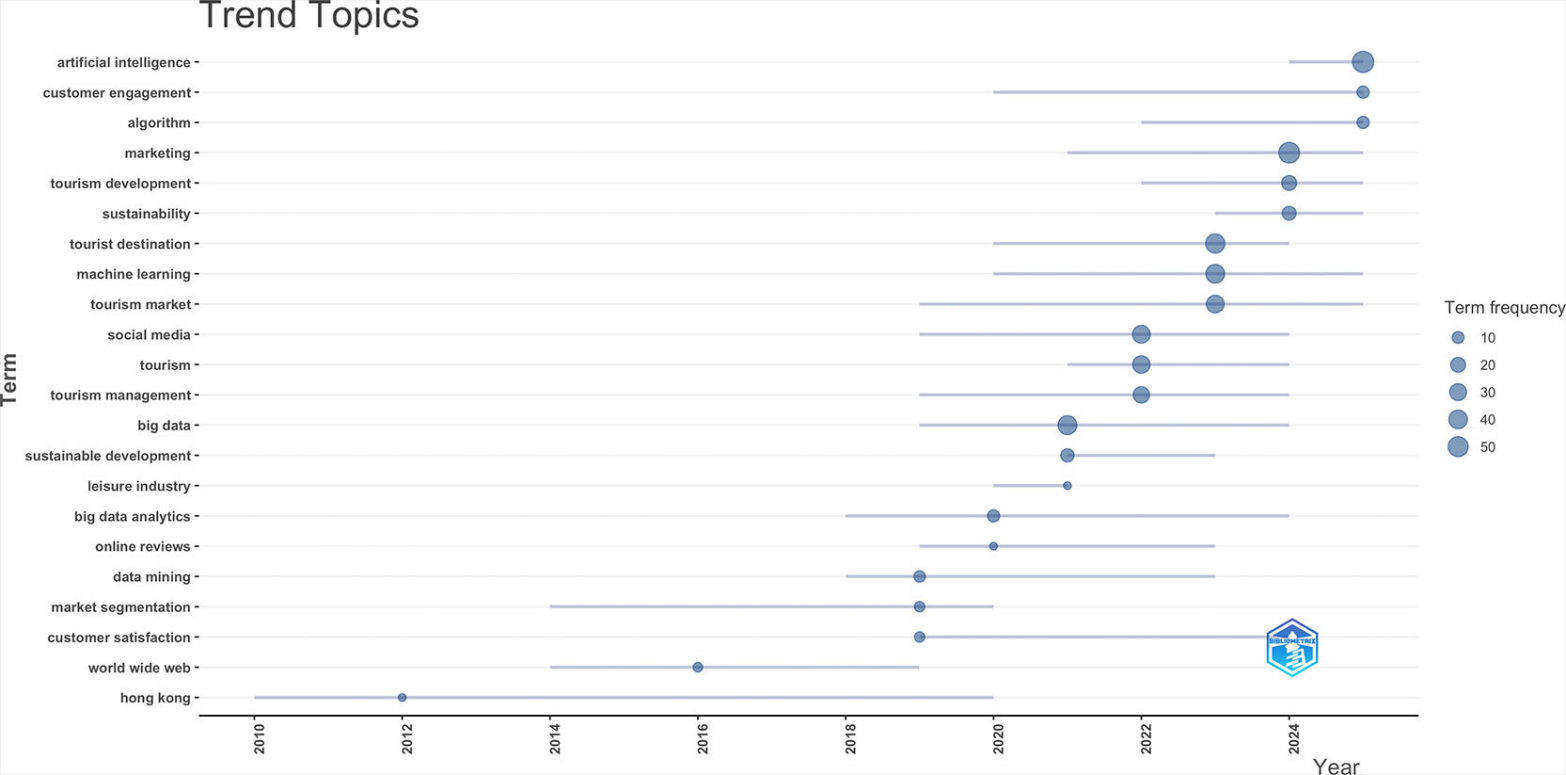
Trend topics (Source: Biblioshiny).

In this subsection, trending topics are examined in terms of authors’ keywords which are explicitly assigned by authors and categorize into the main topical focus for each article ( Song et al., 2019). In Figure 5, a temporal analysis was used to show the development and hierarchical structure of research themes in AI-driven tourism and marketing. Early works (approximately 2010–2014) mainly focused on general topics like tourism and management and online reviews, possibly due to an initial concern for digital sources. Keywords of big data, data mining and social media become increasingly prevalent from 2016 onward, which implies that a transition to the data-intensive paradigm is taking place. Artificial intelligence, machine learning, and algorithms became the keyword also following 2019 with topics associated to technology and advertisement strongly correlated with marketing, destination and customer relations. Furthermore, the increasing emphasis on environmental sustainability and tourism development in recent years coupled with a heightened awareness of customer satisfaction indicates that responsible though experience-focused as well as intelligent forms of tourism marketing are emerging priority areas. Taken all together, the evolution analysis reveals an obvious transformation from descriptive digital tourism to AI-driven analytics-based and strategic-oriented marketing research in the context of tourism.

### 3.6 The thematic map

**Figure 6.**
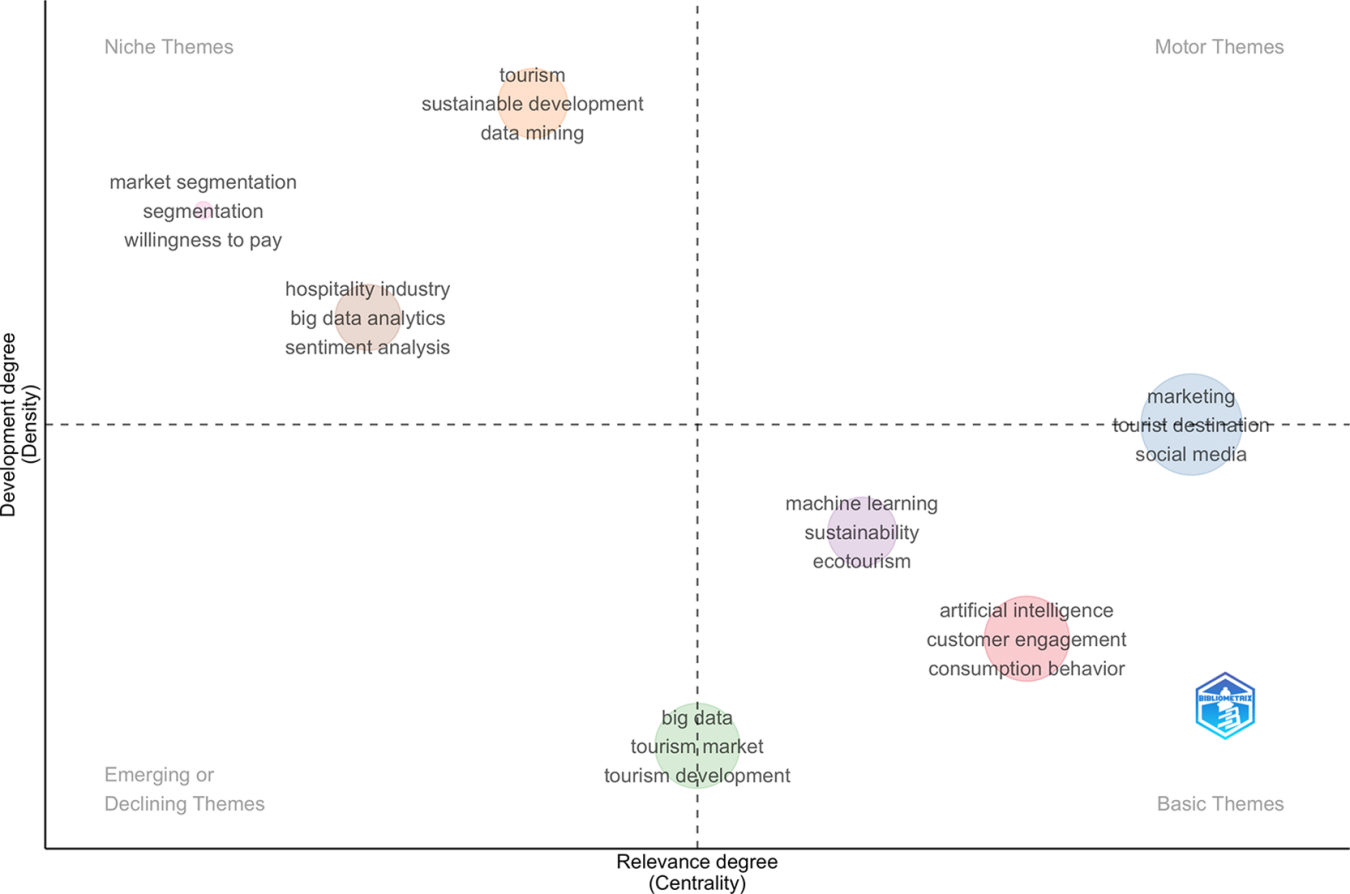
Thematic map (Source: Biblioshiny).

Thematic analysis is critical in informing researchers and other stakeholders of potential paths for the future development of research within thematic areas ( Agbo et al., 2021). The thematic analysis is based on the collation and connecting of authors’ abstracts. There are two characteristics that characterize these themes: (i) centrality and (ii) density. Centrality (on the horizontal axis) quantifies the extent of correlation among various topics, while density (on the vertical axis) assesses the cohesion among nodes. These are the two elements that end up deciding the overall development and relevance of a particular theme. The more edges a node has, the more central and important it becomes with respect to a thematic network. The cohesiveness of a node, which indicates the denseness of a research area, is an indicator of potential to grow and impacts. For the detailed topical summary of AI-enabled tourism and hospitality marketing sector (Q1–Q4), see Figure 6. The upper right quadrant (Quad 1) contains the propelling themes, the lower right quadrant (Quad 4) contains fundamental themes, the upper left quadrant (Quad 2) is concerned with highly specialized themes and finally, the lower left quadrant (Quad 3) is related to emergent or dying off themes:

1. **Motor Themes (Quad 1):** The motor themes quadrant (high centrality and high density) is dominated by marketing, tourist destination, and social media, indicating that these topics are both well-developed and central to the field, and thus act as the main driving forces of current research. Closely related, artificial intelligence, customer engagement, and consumption behavior appear as highly relevant basic themes, suggesting that while these topics are foundational and widely connected to other themes, they continue to evolve and expand in terms of theoretical and methodological depth.
2. **Niche Themes (Quad 2):** The niche themes quadrant includes market segmentation, willingness to pay, and hospitality industry–focused studies, often linked with big data analytics and sentiment analysis. These themes are well developed but remain relatively specialized, addressing specific applications rather than the core of the field.
3. **Emerging/Declining Themes (Quad 3):** The emerging or declining themes quadrant features big data, tourism market, and tourism development, indicating either emerging research directions that have yet to gain strong conceptual integration or mature topics experiencing a gradual decline in scholarly attention.
4. **Basic Themes (Quad 4):** The basic themes quadrant highlights machine learning, sustainability, and ecotourism, reflecting their growing importance and strong connections with the broader research domain, although these themes are still in the process of consolidation.

### 3.7 Bibliographic coupling of countries

**Figure 7.**
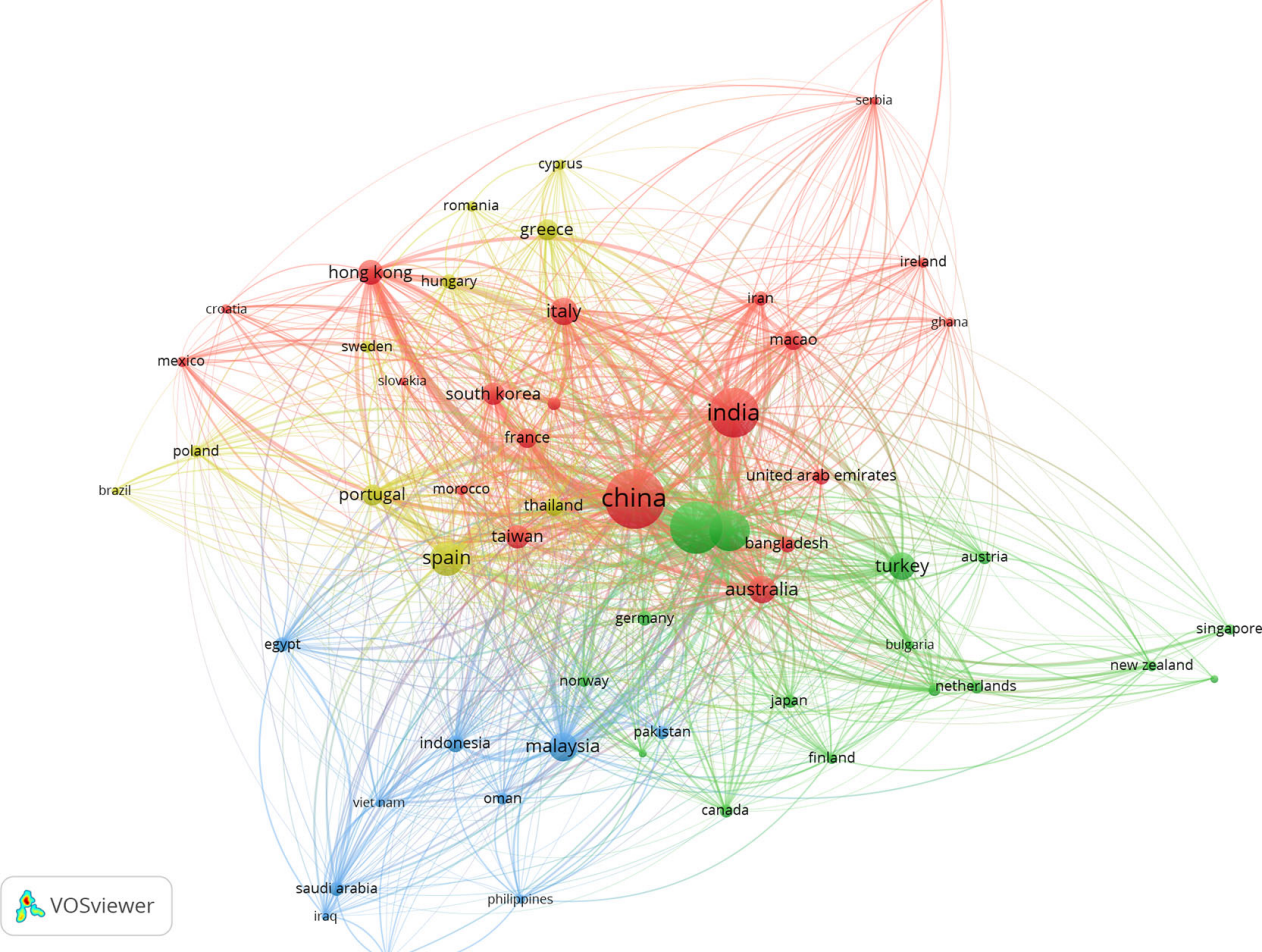
Bibliographic coupling of countries (Source: VOSviewer).

Figure 7 shows the bibliographic coupling of countries, which illustrates the interlink between national research communities through their common references in works on AI in tourism marketing. Countries like China, the U.S., the U.K., Spain, Italy, and Australia are displayed as bigger nodes in the centre of this network suggesting higher research productivity and stronger intellectual links with other countries. These countries cluster closely, indicating a common theoretical and methodological basis to AI, big data marketing in tourism and customer analytics. Conversely, peripheral countries of the network exhibit weaker coupling strengths representing incipient, or more domain-specific research contributions. Taken together, the map reveals a globally networked but clustered research system, with a group of country actors at its core that are pushing the intellectual boundaries -and potential- for more cross-country collaboration.

### 3.8 Bibliographic coupling of documents

**Figure 8.**
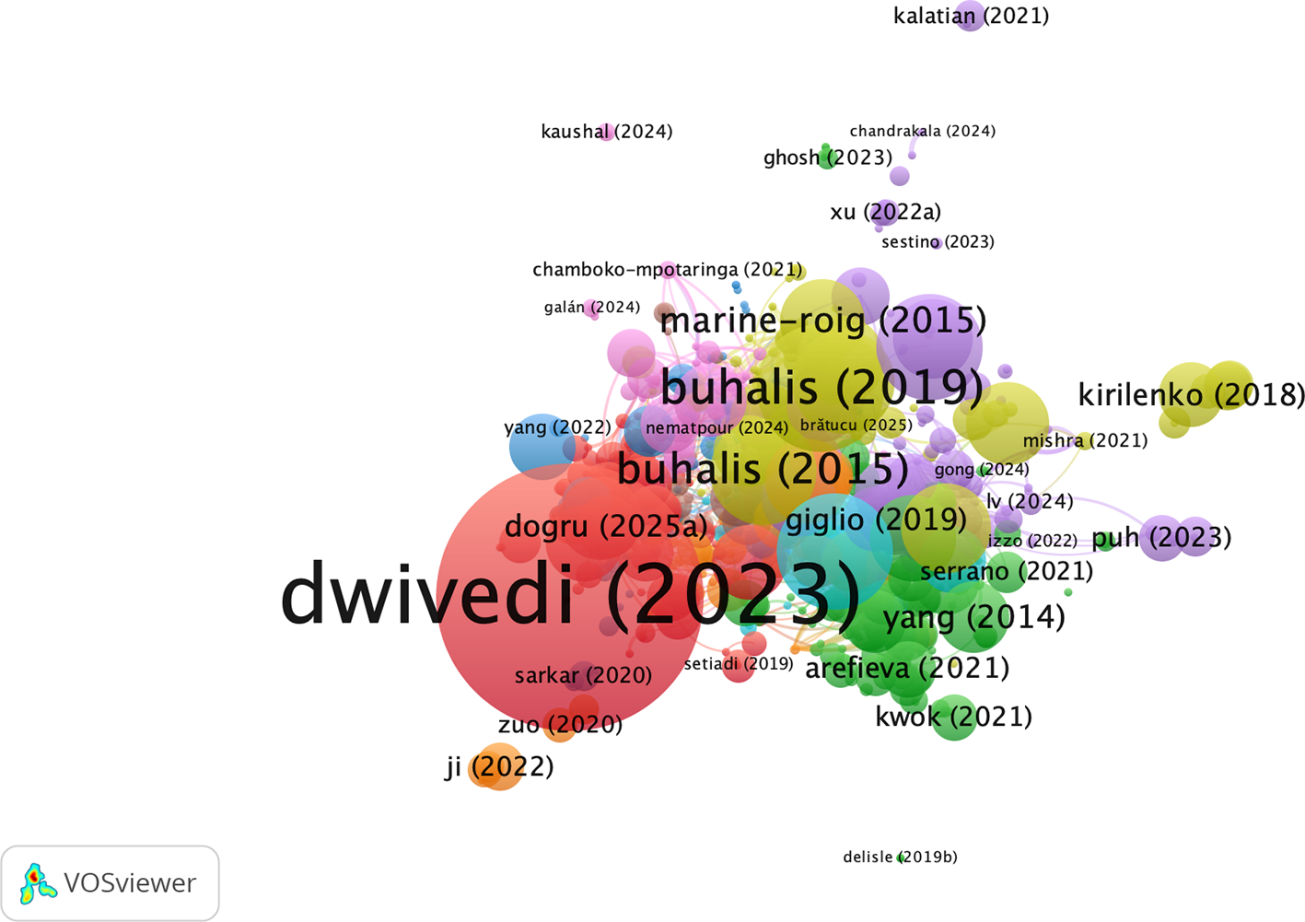
Bibliographic coupling of documents (Source: VOSviewer).

Figure 8 is a citation network map of the most cited documents on AI-based tourism and marketing research created with VOSviewer. The network is clearly led by Dwivedi et al., (2024), who emerges as the tallest and most central node, indicating a high level of impact and the integrative character of his recent AF-ETM publications in AI tourism marketing literature. Seminal contributions of Buhalis et al., (2019), Buhalis & Foerste, (2015) and Marine-Roig & Anton Clavé, (2015), in particular, occupy core locations, emphasizing their informing roles on digital tourism, smart tourism, and technology-enabled destination marketing. In addition, research by Giglio et al., (2019), Yang & Fik, (2014), Kirilenko & Stepchenkova, (2018) and ( Moreno-Luna et al., 2021) composes closely related clusters around these central works to depict thematic consistency around hot topics including big data, social media analytics, tourist behavior, and sustainability. Recent (2021–2024) papers tend to appear in the vicinity of these influential nodes, indicating that current studies can be closely linked to previous theories and methodologies. In sum, the network unveils a coherent and cumulative structure of knowledge development in which a set of a few strongly cited seminal papers stakes out territory after which AI applications in tourism and hospitality marketing are extended or refined by younger works.

### 3.9 Thematic analysis using keywords co-occurrence network

**Figure 9.**
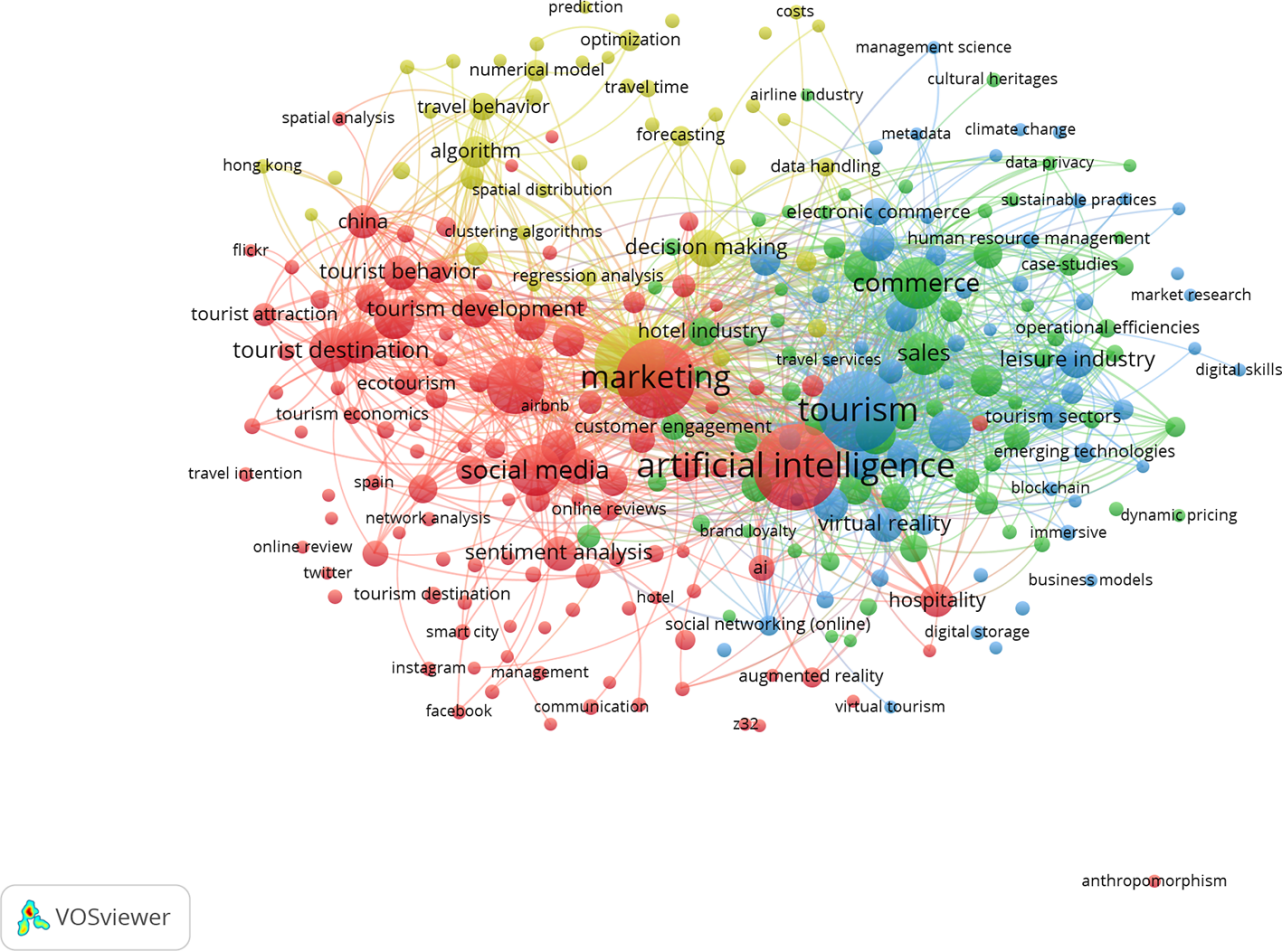
Keywords co-occurrence network (Source: VOSviewer).

This section examines the thematic structure of research on artificial intelligence–enabled tourism and marketing through a keywords co-occurrence network analysis conducted using VOSviewer. Author-defined keywords were analysed to identify frequently co-occurring terms, enabling the detection of dominant research themes and their interrelationships. As illustrated in Figure 9, the network visualization reveals distinct clusters based on the strength of keyword associations, reflecting the intellectual organization and evolving focus areas of the field. To facilitate interpretation, the identified clusters were grouped into four major thematic domains, each representing a coherent stream of research. Table 2 summarizes these clusters by highlighting their corresponding colours, theme names, core research focus, and representative keywords, thereby providing a structured overview of the key thematic directions shaping AI-driven tourism and hospitality marketing literature.

**Table 2.** Keywords co-occurrence network summary table.

Cluster colour	Theme name	Core focus	Representative keywords
**Red**	Digital Influence and Tourist Behaviour Analytics	Examines the role of social media in shaping tourist perceptions and travel intentions using data analytics and sentiment analysis.	Social media, tourist destination, tourist behavior, sentiment analysis, online reviews, customer engagement, Instagram, Twitter, tourism development
**Green**	AI-Enabled Smart Tourism and Commerce Ecosystems	Focuses on the integration of artificial intelligence with tourism operations, commerce, sustainability, and emerging digital technologies to enhance value creation and efficiency.	Artificial intelligence, tourism, commerce, sales, leisure industry, sustainable practices, blockchain, pricing, emerging technologies
**Blue**	Technology-Driven Hospitality and Experience Innovation	Explores the use of immersive and digital technologies to transform hospitality services, customer experience, and tourism business models.	Hospitality, virtual reality, augmented reality, immersive tourism, business models, digital transformation, operational efficiencies
**Yellow**	Data-Driven Decision Making and Predictive Tourism Modelling	Addresses the application of algorithms, machine learning, and predictive analytics for forecasting, optimization, and strategic decision-making in tourism management.	Algorithms, prediction, forecasting, optimization, decision making, numerical models, spatial analysis, airline industry

Source: Author’s own.

***Theme 1: Digital Influence and Tourist Behaviour Analytics (Red Cluster)***

Digital platforms, social media, and data analytics now shape how tourists choose destinations, move, spend, and share experiences. Research combines psychological models with big data and AI to understand and predict these behaviours for smarter tourism management. Destination online content (TDOC) quality and user-friendly accessibility strongly shape intentions to visit and eWOM; satisfaction mediates these effects but may affect sharing more than revisits ( Armutcu et al., 2023). Social media attributes such as source credibility, homophily, and content quality build destination image and trust, driving impulsive travel intentions ( Wei et al., 2025). Technology acceptance factors (usefulness, ease of use, interactivity) foster positive attitudes toward social media and influence travel behaviour (W. Chen et al., 2024). Gen X values personal recommendations and reliability; Gen Y/Z rely more on online reviews, social media, visual and real-time content, with mobile-first decision-making ( Mikhailov & Kashevnik, 2020). Geo-tagged photos, taxi GPS, and social media check-ins are mined to model movement patterns, popular zones, and flows for destination management ( Mikhailov & Kashevnik, 2020). Clustering, fuzzy clustering, and ontological “digital pattern of life” models segment tourist types and predict preferences over time ( Mikhailov & Kashevnik, 2020). Neural networks and Bayesian networks improve prediction of spending behaviour, demand, and resource allocation, outperforming traditional models ( Mikhailov & Kashevnik, 2020; Tian & Tang, 2025). Digital influence on tourist behaviour is substantial: high-quality, usable content; credible influencers; and engaging, mobile-friendly social media shape where people go, how they spend, and what they share. At the same time, big data and AI techniques enable fine-grained prediction of movement and spending, supporting more targeted marketing, smarter infrastructure, and improved visitor experience.

***Theme 2: AI-Enabled Smart Tourism and Commerce Ecosystems (Green Cluster)***

AI is reshaping tourism into connected “smart” ecosystems that blend destinations, services, and commerce into seamless, personalized journeys. Across recent work, the focus is on integrating AI with IoT, cloud, and platform models to optimize experience, sustainability, and monetization. Machine learning, NLP, and computer vision process IoT sensor data to enable real-time monitoring, demand forecasting, and context-aware services in Smart Tourism Destinations (STDs) ( Aliyah et al., 2023). Integrated GenAI–NLP–IoT systems support trip planning, predictive analytics, multilingual dialogue, and accessibility (audio/voice for disabled tourists) in real-world pilots ( Suanpang & Pothipassa, 2024). Foundation-model–based “Parallel Tourism Systems” link virtual simulations with real services, governed by DAOs, to offer agentic, autonomous, end-to-end travel support (). AI-driven marketing in smart destinations supports hyper-personalized offers, SoCoMo campaigns, and revenue optimization while attracting investment and improving residents’ quality of life ( Huang et al., 2025). Across tourism and e-commerce, recommendation engines, conversational agents, dynamic pricing, and fraud detection turn journeys into continuous, data-driven engagement loops ( Erdős et al., 2025). Systematic reviews highlight AI-enabled customer experience (AICX) as a distinct construct, stressing customer-facing touchpoints and human–AI balance (Y. Chen & Prentice, 2025). AI-enabled smart tourism and commerce ecosystems integrate AI, IoT, cloud, and platform governance to deliver personalized, real-time, and sustainable services while optimizing revenue and operations. The opportunity is large, but success depends on ethical data practices, transparency, inclusivity, and preserving meaningful human experiences in increasingly automated environments.

***Theme 3: Technology-Driven Hospitality and Experience Innovation (Blue Cluster)***

Digital technologies are reshaping how hospitality firms design, deliver, and manage guest experiences, from mobile apps and AI to VR, robotics, and IoT. Research shows that the most successful innovations blend high-tech with high-touch, using technology to personalize, de-risk, and streamline experiences while preserving the human “soul” of hospitality. Smart environments (IoT, AI, VR/AR) enable extra-sensory, hyper-personalized, and beyond-automation experiences in tourism and hospitality ( Buhalis et al., 2019). Digital hospitality includes VR, contactless tech, AI, mobile apps, and cloud systems that reconfigure service encounters and operations ( Buhalis et al., 2019). Human-related service innovation has a stronger impact on satisfaction and delight than purely technology-related innovation; technology best works as a moderator/amplifier of human service ( Buhalis et al., 2019). Frameworks emphasize a human-centered, experience-oriented approach where technology frees staff for emotional engagement and relationship-building ( Kandampully et al., 2023). Luxury and upscale hotels use “techno-business strategies” to balance state-of-the-art tech with personalized, unscripted service tailored to micro-segments ( Kandampully et al., 2023). Research gaps around cocreative technologies, HR and change management, strategy, and digital transformation remain. Barriers include implementation cost, resistance to change, and maintaining authenticity and privacy ( Neuhofer et al., 2015). Future agendas call for context-specific studies, sustainable digital hospitality, and deeper integration of people-centric design with emerging tech ecosystems ( Park et al., 2023; Tai et al., 2021). Technology-driven hospitality innovation is most effective when it enhances personalization, safety, and efficiency without displacing the human connection that defines memorable stays. Evidence supports a “high-tech, high-touch” model where smart technologies enable richer co-created experiences, while strategic, human-centered innovation safeguards trust, emotion, and the enduring “soul” of hospitality ( Roy & Pagaldiviti, 2023).

***Theme 4: Data-Driven Decision Making and Predictive Tourism Modelling (Yellow Cluster)***

Data-driven decision making and predictive modelling now underpin how destinations, hotels, and policymakers plan capacity, manage risk, and design tourism strategies. Research spans big data analytics, AI/machine learning forecasting models, and organizational adoption of data-centric cultures. AI and deep learning models (e.g., deep networks, decomposed deep learning, BiLSTM–Transformer hybrids) significantly outperform traditional econometric/time-series models in forecasting tourist arrivals and demand, while also revealing key drivers of demand ( Dimitriadou et al., 2025; Hu et al., 2025). Machine learning algorithms such as SVM, Random Forest, Gradient Boosting, and mixed-frequency BiLSTM-MIDAS improve accuracy for arrivals, hotel occupancy, keyword trends, and cross-country demand under uncertainty ( Dimitriadou et al., 2025; Hu et al., 2025). Big data sources (search queries, social media, geotagged photos, UGC) support fine-grained prediction of flows, preferences, and behaviors at destinations ( Li et al., 2021). Recent work pushes hybrid AI models, mixed-frequency data, and uncertainty measures; however, integration of analytics capabilities, governance, and sustainability indicators into routine strategic decisions remains underdeveloped. Data-driven tourism now relies heavily on AI, machine learning, and big data to predict demand, behavior, and financial trends, consistently beating traditional models in accuracy. The main frontier is less technical than organizational: building data cultures, governance, and skills so destinations and firms can embed these predictive tools into every day, ethical, and sustainability-oriented decision making ( Mu et al., 2025; Zhang et al., 2021).

## 4. Conclusion

This holistic bibliometric and thematic review exhaustively mapped the intellectual domain of AI-enabled tourism and hospitality marketing research by analyzing 320 peer-reviewed publications published between 2003 to 2025. The paper uncovers exponential growth in articles since 2017. The bibliometric study reveals a mature but evolving knowledge domain formed on the depth of work from early scholars like Buhalis, whose research has been central in developing concepts for technology and tourism. The study also reveals intellectual leadership exerting from China, followed by the United States, UK, Spain, Italy and Australia. The thematic analysis through keyword co-occurrence networks uncovered four distinct yet interconnected research clusters: (1) Digital Influence and Tourist Behaviour Analytics, (2) AI-Enabled Smart Tourism and Commerce Ecosystems, (3) Technology-Driven Hospitality and Experience Innovation, and (4) Data-Driven Decision Making and Predictive Tourism Modelling. These themes collectively define the current state and future trajectory of the field. The temporal analysis reveals a clear evolutionary trajectory from early descriptive studies of digital tourism and online reviews toward sophisticated AI-powered analytics, predictive modeling, and sustainability-oriented marketing approaches. Recent literature increasingly addresses critical ethical considerations including data privacy, algorithmic transparency, consumer trust, and the delicate balance between automation efficiency and human-centric service delivery.

## 5. Future research directions

The future research directions are illustrated in Table 3.

**Table 3.** Future research directions.

S.No	Cluster	Future scope
**1**	**Cluster 1 - Red: Digital Influence and Tourist Behaviour Analytics**	Examine how tourists consume information and content from social media platforms (e.g., Instagram, TikTok, YouTube, WeChat) and the role of platform-specific features on destination image formation, travel intentions’ generation and booking decision making. Conduct longitudinal studies examining multigenerational reactions to AI-generated personalized marketing communications, influencer content and user-generated reviews giving consideration to the influence of digital provides on cultural and technological literacy. Create state-of-the-art deep neural network structures for real-time multimodal (geo-locale, social media sentiment, weather, local events) tourist-behaviour prediction to enable dynamic destination management and crisis response.
**2**	**Cluster 2 - Green: AI-Enabled Smart Tourism and Commerce Ecosystems**	Design the overall governance frameworks for AI-driven smart tourism ecosystems, accommodating the conflicting interests of different stakeholders (tourists, companies, citizens and governments) between ROI (Return on Investment) of using AI technology; data sovereignty as well as fair value distribution. Examine the relationship between blockchain technology and AI system for transparent & secure decentralized tourism commerce system that demonstrate loyalty behavior, authenticity certification of experiential tour program or peer-to-peer service exchange in this pioneering path. Analyse how AI-IoT-powered integrated systems can efficiently optimise resource levels, such as energy and water utilisation, carbon footprints and overtourism; influence sustainable tourist behaviours through real-time monitoring/predictive analytics/nudging.
**3**	**Cluster 3 - Blue: Technology-Driven Hospitality and Experience Innovation**	Discover the emergent phenomenon of metaverse tourism —virtual travel experiences, digital destinations and hybrid physical-virtual tourism—from business model, experience design principles & impact on traditional travel industries. Analyze for comparison the dynamics of interaction between humans and service robots chatbot a AI concierge at hospitality: impact on tourists’ acceptance, satisfaction and emotional attachment respectively to service robot, chatbot and AI concierge. Investigate how technology-mediated experiences affect genuine relationship building, cultural understanding and meaningful engagement with a particular description on the discussion of the tension between emergent technology experiences and place-based authenticity.
**4**	**Cluster 4 - Yellow: Data-Driven Decision Making and Predictive Tourism Modelling**	Create XAI methodologies for tourism forecasting models, recommendation algorithms and price decisions to become transparent and interpretable by managers, ensuring trustworthiness, regulatory compliance and actionable insights. Build specialist algorithms to forecast low-frequency, high-impact events in travel (political upheavals, pandemics, natural catastrophes, viral social media moments) that significantly impact demand but which are hard to predict with traditional methods.

Source: Author’s own.

## 6. Implications

This systematic review makes several important theoretical contributions by providing a comprehensive intellectual structure of the field, identifying knowledge gaps and underexplored areas, and establishing a baseline for understanding the maturation of AI applications in tourism. From a practical standpoint, the findings offer actionable insights for tourism practitioners, hospitality managers, destination marketing organizations, and technology vendors. In conclusion, AI-based tourism and hospitality marketing has matured into a vibrant, multidisciplinary field characterized by robust theoretical foundations, diverse methodological approaches, and significant practical implications. As the field continues to evolve, maintaining a balanced focus on technological innovation, ethical responsibility, sustainability, and human-centric design is required for preserving the authentic, transformative essence of travel.

## 7. Limitations

This study, like others, possesses limitations that suggest new directions for research. The data was first acquired from the Scopus database, predominantly from journals and scholarly papers. Given the constraints of the current research, subsequent studies may explore alternate conceptualizations by utilizing data from additional pertinent sources, like Web of Science and EBSCOhost. Secondly, the dataset comprised exclusively papers published in English, which were identified using specific keywords in the Scopus database. Future investigations may yield more comprehensive results by employing different keywords and examining research published in various languages.

## Ethics and consent

Ethics and consent not required for this study.

## Data Availability

**Zenodo:** Mapping Research Trends in AI-Based Tourism and Hospitality Marketing: A Bibliometric and Thematic Review, https://doi.org/10.5281/zenodo.18296037 ( Tyagi & Singh, 2026). This project contains the following underlying data: 1. Data Set.csv Data are available under the terms of the Creative Commons Attribution 4.0 International license (CC-BY 4.0).
