# Supplementary material for: Mapping Research Trends in AI-Based Tourism and Hospitality Marketing: A Bibliometric and Thematic Review
Source: F1000Res. 2026 Apr 20;15:192. Originally published 2026 Feb 5. [Version 2] doi: 10.12688/f1000research.177254.2 (PMC12936479; doi:10.12688/f1000research.177254.2)
Supplement: Peer review history

# Reviewer report: Reviewer response for version 2

**DOI:** 10.5256/f1000research.197482.r476749

**Published:** 2026-06-08

**Authors:** Aditya Ranjan

**Affiliations:** 1 ITM University, Gwalior, Madhya Pradesh, India

The authors have addressed the major and minor suggestions made.

Is the work clearly and accurately presented and does it cite the current literature?

Yes

If applicable, is the statistical analysis and its interpretation appropriate?

Not applicable

Are all the source data underlying the results available to ensure full reproducibility?

Yes

Is the study design appropriate and is the work technically sound?

Yes

Are the conclusions drawn adequately supported by the results?

Yes

Are sufficient details of methods and analysis provided to allow replication by others?

Yes

Reviewer Expertise:

Tourism, Spiritual Tourism

I confirm that I have read this submission and believe that I have an appropriate level of expertise to confirm that it is of an acceptable scientific standard.

# Reviewer report: Reviewer response for version 1

**DOI:** 10.5256/f1000research.195448.r456307

**Published:** 2026-02-25

**Authors:** Nagendra Yadav

**Affiliations:** 1 Welcomgroup Graduate School of Hotel Administration, Manipal Academy of Higher Education (Ringgold ID: 177107), Manipal, Karnataka, India

**SUMMARY OF THE ARTICLE**

 This manuscript presents a comprehensive bibliometric and thematic analysis of artificial intelligence applications in tourism and hospitality marketing, analysing 320 peer-reviewed articles from Scopus database spanning 2003 to 2025. Using the SPAR-4-SLR protocol combined with VOSviewer and Biblioshiny software, the authors map the intellectual structure, publication trends, influential contributions, and thematic evolution of the field. The study identifies exponential publication growth post-2017, highlighting China, USA, UK, Spain, Italy, and Australia as leading research hubs, with Buhalis emerging as a seminal contributor. Through keyword co-occurrence analysis, four interconnected thematic clusters are revealed: Digital Influence and Tourist Behaviour Analytics; AI-Enabled Smart Tourism and Commerce Ecosystems; Technology-Driven Hospitality and Experience Innovation; and Data-Driven Decision Making and Predictive Tourism Modelling.

**REVIEW REPORT**

**Question 1: Clarity, Accuracy, and Literature Review - Yes**

 The manuscript demonstrates a strong command of bibliometric methodology literature and effectively contextualises AI's transformative impact on tourism marketing with recent citations (2023-2025) demonstrating currency. However, critical issues require attention. The introduction explicitly states "three research questions" but only presents RQ1 and RQ2, with RQ3 never articulated. This fundamental structural flaw must be corrected. A corrupted citation "[()] {mark}" appears in Theme 2, compromising scholarly integrity and requiring immediate correction.

**Question 2: Study Design and Technical Soundness - Yes**

 The SPAR-4-SLR protocol provides a rigorous, systematic framework appropriate for bibliometric reviews, with a well-structured three-stage methodology following established best practices. The integration of bibliometric and thematic analysis offers complementary insights, Scopus selection is justified, and the 23-year timespan appropriately captures field evolution. While single-database limitation and grey literature exclusion are noted, these are acknowledged in limitations and acceptable given resource constraints.

**Question 3: Methods and Replicability - Yes**

 The methodology provides excellent transparency, enabling independent replication. The search strategy is comprehensively documented with specific Boolean operators. Inclusion/exclusion criteria are explicit, covering document types, subject areas, and language restrictions. The purification process transparently explains the systematic exclusion of 1,908 documents. Software specifications are provided (VOSviewer 1.6.8, Biblioshiny). Most impressively, the complete dataset is deposited in Zenodo (DOI: 10.5281/zenodo.18296037) under Creative Commons Attribution 4.0 license in accessible CSV format, representing gold-standard open science practice enabling full independent verification.

**Question 4: Statistical Analysis - Not applicable**

 The analytical approaches, frequency analysis, bibliographic coupling, keyword co-occurrence networks, temporal trends, and thematic mapping are entirely appropriate for exploratory bibliometric research objectives and represent established methodological practices.

**Question 5: Data Availability and Reproducibility - Yes**

 Exemplary standard achieved. Full dataset openly deposited in Zenodo with permanent DOI under Creative Commons Attribution 4.0 license in universally accessible CSV format. This comprehensive data sharing enables verification, replication, and extension reproducibility. The combination of transparent methodology, detailed search documentation, and open data represents best practice in contemporary bibliometric research.

**Question 6: Validity of Conclusions - Yes**

 This manuscript represents high-quality bibliometric scholarship with rigorous methodology, comprehensive analysis of 320 publications, and exemplary data transparency. The SPAR-4-SLR framework is appropriately applied, the four thematic clusters provide valuable intellectual structure, and the temporal analysis effectively documents field evolution. The Zenodo data deposit represents a gold-standard reproducibility practice. The work makes meaningful theoretical contributions by synthesising fragmented literature and identifying research trajectories. Minor typographical refinements during copyediting would enhance polish, but the manuscript is scientifically sound and suitable for publication. This review will serve as a valuable reference for researchers, practitioners, and policymakers navigating AI-tourism marketing intersections.

**Strengths of the Study**

**Methodological Rigor: **The study employs a well-structured SPAR-4-SLR protocol combined with bibliometric analysis using VOSviewer and Biblioshiny, which is appropriate for mapping research trends. The systematic approach with clear inclusion/exclusion criteria (filtering from 2,228 to 320 documents) enhances reproducibility and transparency.

**Comprehensive Coverage: **The analysis spans 23 years (2003-2025) and identifies four distinct thematic clusters: (i) Digital Influence and Tourist Behaviour Analytics; (ii) AI-Enabled Smart Tourism and Commerce Ecosystems; (iii) Technology-Driven Hospitality and Experience Innovation; and (iv) Data-Driven Decision Making in Predictive Tourism Modelling. This provides a holistic view of the field's evolution.

**Clear Structure and Presentation: **The manuscript follows a logical progression from introduction through methodology to findings, with well-organized sections and systematic presentation of bibliometric results including publication trends, influential authors, and country-level contributions.

**Major Improvement**

1. Fix the research-question logic: the Introduction says, “three research questions,” but only RQ1 and RQ2 are shown; either add the missing RQ3 explicitly (and ensure results address it) or revise the text to “two research questions.”
2. Resolve the “sentiment analysis” gap: the Methods state that sentiment analysis in R was performed, but the Findings sections you present are overwhelmingly bibliometric/thematic; either (a) add a clear sentiment-analysis subsection (data inputs, method/lexicon/model, outputs, validity checks) and integrate it into results, or (b) remove/soften the claim if it wasn’t actually executed/reported.

**Minor Improvement**

1. Some references appear incomplete or inconsistently formatted. A thorough proofread against the journal citation guidelines is needed. Several sentences require revision for clarity and grammatical accuracy.

Is the work clearly and accurately presented and does it cite the current literature?

Yes

If applicable, is the statistical analysis and its interpretation appropriate?

Yes

Are all the source data underlying the results available to ensure full reproducibility?

Yes

Is the study design appropriate and is the work technically sound?

Yes

Are the conclusions drawn adequately supported by the results?

Yes

Are sufficient details of methods and analysis provided to allow replication by others?

Yes

Reviewer Expertise:

Hospitality & Tourism

I confirm that I have read this submission and believe that I have an appropriate level of expertise to confirm that it is of an acceptable scientific standard.

# Reviewer report: Reviewer response for version 1

**DOI:** 10.5256/f1000research.195448.r456302

**Published:** 2026-02-25

**Authors:** Aditya Ranjan

**Affiliations:** 1 ITM University, Gwalior, Madhya Pradesh, India

**SUMMARY**

 This manuscript presents a systematic bibliometric and thematic review examining the intellectual landscape of artificial intelligence (AI) applications in tourism and hospitality marketing. The authors analyzed 320 peer-reviewed publications from the Scopus database spanning 2003-2025, employing the SPAR-4-SLR protocol alongside VOSviewer and Biblioshiny software for bibliometric analysis. The study addresses two primary research questions: (1) What is the current state of research in AI-based tourism and hospitality marketing? (2) What are the emerging themes in this domain?. The analysis reveals exponential growth in publications since 2017, with China, the United States, UK, Spain, Italy, and Australia emerging as intellectual leaders. Through keyword co-occurrence network analysis, four distinct thematic clusters were identified: Digital Influence and Tourist Behaviour Analytics, AI-Enabled Smart Tourism and Commerce Ecosystems, Technology-Driven Hospitality and Experience Innovation, and Data-Driven Decision Making and Predictive Tourism Modelling. The manuscript contributes to theoretical understanding by mapping the field's evolution from descriptive digital tourism studies toward sophisticated AI-powered analytics and sustainability-oriented marketing approaches.

**1. Clarity, Accuracy, and Literature Review**

 The manuscript is clearly and accurately presented. The authors have structured the paper logically, making it easy for the reader to follow the progression of the study. The language is precise, and the key concepts related to AI in tourism marketing are well explained. The literature review is comprehensive, citing a wide range of relevant studies from foundational research to more recent publications. The references are up-to-date and cover a broad spectrum of topics within the field of AI and tourism marketing. The review appropriately highlights the emerging trends, methodologies, and key areas of research. However, the manuscript could benefit from a more detailed discussion of the limitations or potential biases in the existing literature to provide a more balanced view.

**Suggestion**: The authors may consider adding a brief section on the limitations of the studies reviewed, particularly with regard to study methodologies, sample sizes, and potential biases that could influence the findings.

**2. Study Design and Technical Soundness**

 The study design is appropriate for the research objectives. The authors utilize a systematic approach, adhering to the SPAR-4-SLR protocol, which is a recognized methodology for conducting systematic literature reviews. The use of bibliometric analysis tools like VOSviewer and Biblioshiny is technically sound, and these tools are well-suited for identifying trends, publication patterns, and thematic clusters within the literature. The design allows the authors to answer their research questions comprehensively, offering both descriptive and relational insights into the field. The methodology is robust, ensuring the reliability of the results.

**3. Methods and Replicability**

 The authors provide sufficient details regarding the methodology used in the study, allowing for full replication by other researchers. The process for selecting the studies from the Scopus database is clearly explained, as are the inclusion and exclusion criteria. The use of VOSviewer and Biblioshiny for bibliometric analysis, as well as the thematic analysis through keyword co-occurrence networks, is well-described. The clear description of the search strategy and the tools used makes it possible for others to replicate the study.

**Suggestions**: The manuscript could be strengthened by providing more information on the exact search terms and Boolean operators used in the query would help future researchers replicate the study more easily.

**4. Statistical Analysis (if applicable)**

 The study does not involve traditional statistical analysis, as the focus is on bibliometric and thematic analysis using specialized software tools like VOSviewer and Biblioshiny. These tools provide visual insights into the relationships and trends within the literature, but no formal statistical hypothesis testing is performed. Given the nature of the research, this absence of statistical analysis is appropriate.

**5. Data Availability and Reproducibility**

 The data underlying the study is fully available, ensuring reproducibility. The authors have made their dataset accessible via Zenodo, which includes the underlying research papers and data used in the bibliometric analysis. This enhances the transparency of the research and allows others to verify or build upon the findings. The detailed explanation of the data retrieval process from Scopus and the associated tools used ensures that the results can be independently reproduced.

**6. Validity of Conclusions**

 The conclusions drawn in the manuscript are adequately supported by the results. The authors provide a well-founded synthesis of the trends and thematic clusters within AI-based tourism and hospitality marketing, and their conclusions align with the findings presented in the study. The thematic clusters identified (such as digital influence, AI-enabled smart tourism, and predictive tourism modeling) are well-supported by the bibliometric and thematic analyses conducted. The study also highlights the growing importance of AI in the field, drawing attention to emerging trends and research areas.

**Suggestions**: While the conclusions are well-supported, the authors could further discuss the limitations of their study, particularly regarding the exclusion of non-English articles and the potential biases introduced by the search strategy. This would add transparency and provide a more nuanced understanding of the study's findings.

**Major Suggestions**

**1: Missing Research Question**

 The Introduction explicitly states "the aims of this article are to answer three research questions (RQs)" but subsequently presents only two questions (RQ1 and RQ2). RQ3 is never articulated. This creates ambiguity about the research scope and leaves readers questioning whether analysis is incomplete or if a typographical error occurred.

**2. Incomplete Citation**

 In Theme 2 (Green Cluster), the text references "Parallel Tourism Systems" followed by an incomplete citation: "[()]" without author or year.

**Minor Suggestion**

**1: Acronym Definitions**

- Define "CLV" on first use
- Expand "SoCoMo" 
- Clarify "TDOC": 

**2: Citation Format Consistency**

 Review the entire manuscript to ensure uniform citation style.

Is the work clearly and accurately presented and does it cite the current literature?

Yes

If applicable, is the statistical analysis and its interpretation appropriate?

Not applicable

Are all the source data underlying the results available to ensure full reproducibility?

Yes

Is the study design appropriate and is the work technically sound?

Yes

Are the conclusions drawn adequately supported by the results?

Yes

Are sufficient details of methods and analysis provided to allow replication by others?

Yes

Reviewer Expertise:

Tourism, Spiritual Tourism

I confirm that I have read this submission and believe that I have an appropriate level of expertise to confirm that it is of an acceptable scientific standard.
